# Real-world study of adverse events associated with sodium zirconium cyclosilicate based on FDA adverse event reporting system and VigiAccess database

**DOI:** 10.1371/journal.pone.0333692

**Published:** 2025-10-10

**Authors:** Xiaona Jia, Lei Liu, Pan Wang

**Affiliations:** Department of Pharmacy, Civil Aviation General Hospital, Beijing, China; Kwara State University, NIGERIA

## Abstract

The aim of this study was to investigate frequencies, types, and signals of adverse drug events (ADEs) associated with sodium zirconium cyclosilicate (SZC) used for the treatment of hyperkalemia, in order to inform clinicians of possible safety concerns linked with SZC in real-life usage. ADE reports associated with SZC were collected from both the FAERS and VigiAccess databases. Data extraction from FAERS was performed using OpenVigil 2.1, covering reports from the first quarter of 2004 through the third quarter of 2024. The VigiAccess database was retrieved for reports up to February 5, 2025. The ADEs were standardized and classified by using the preferred term (PT) and the system organ class (SOC) of the Medical Dictionary for Regulatory Activities (MedDRA) (Version 27.0). The reporting odds ratio (ROR) method and the proportional reporting ratio (PRR) method were used to screen positive signals and analyze the characteristics of ADE signals. In this study, 1384 and 1518 ADE reports related to SZC were obtained from the FAERS database and the VigiAccess database, respectively. At the SOC level, the ADEs retrieved in the two databases involved 26 SOCs, and the top 3 SOCs in terms of the number of reported cases were general disorders and administration site conditions, gastrointestinal disorders, and investigations. At the PT level, among the top 30 PTs in terms of the number of reported cases in the two databases, death, cardiac failure, weight increased, blood pressure increased, cardiac failure congestive, cerebrovascular accident, myocardial infarction, pneumonia, dizziness, dysphagia, and dyspnoea were the ADEs with higher number of reported cases not included in the drug instructions. A total of 41 positive signals were obtained after signal screening in FAERS database. Among them, the top 3 PTs in terms of signal strength were blood potassium abnormal (ROR = 180.224[119.925, 270.842]), blood potassium increased (ROR = 98.789[78.835, 123.792]), blood sodium increased (ROR = 35.248[14.624, 84.961]). Signals of cardiac disorders such as cardiac failure chronic, cardiac failure and cardiac failure congestive, signals of gastrointestinal disorders such as ileus and intestinal perforation, and signals of blood sodium increased and hypernatraemia are positive signals that deserve special attention. In this study, the common ADEs associated with SZC were confirmed, and several intriguing novel signals not included in the drug instructions were discovered, which would provide more safety reference data for the clinical use of SZC.

## Introduction

Hyperkalemia is a condition in which serum potassium ions (K+) exceed 5.0 mmol/L. It is a common electrolyte disorder, particularly among specific patient populations such as patients with chronic kidney disease (CKD), diabetes, or heart failure, and those receiving renin-angiotensin-aldosterone system inhibitors (RAASis) [1,2]. This disease can lead to life-threatening conditions such as severe cardiac arrhythmias and sudden death [3–6]. Previous pharmaceutical interventions for the treatment of hyperkalemia were mainly organic polymer resins such as sodium polystyrene sulfonate (SPS), but it showed no selectivity for K+, resulting in poor tolerance and/or ineffectiveness [7]. Sodium zirconium cyclosilicate (SZC) is a novel oral potassium-lowering pharmaceutical agent. It is an inorganic, insoluble, and highly selective K+ binding agent that exchanges sodium and hydrogen ions for K+ or ammonium ions in the gastrointestinal tract, thereby increasing fecal potassium excretion and reducing serum potassium levels [8]. SZC demonstrated significantly greater adsorption capacity for K+ compared to other ions. And it has no impact on the utilisation of RAASis drugs. Additionally, SZC demonstrated a low propensity to induce electrolyte disorders in the body and showed no significant effect on liver function, nutritional status and other indicators, showing good tolerability and safety [4,9,10]. The SZC was first approved for the treatment of hyperkalemia in adults in the European Union and the United States in 2018, and then marketed in China at the end of 2019, and is now widely recommended and used [11,12]. Although some adverse reactions of SZC have been reported in previous clinical trial studies [13–16], there is still a lack of research on adverse reactions based on real-world data after being widely used on the market. Clinical trial studies limited by sample size and follow-up time may underestimate the incidence of infrequent or severe adverse drug events (ADEs). The VigiAccess database is a database used by the World Health Organization (WHO) to collect global adverse drug events. The Food and Drug Administration Adverse Event Reporting System (FAERS) database of the United States contains adverse event data for a series of drugs marketed in the United States [17–19]. In this study, we mined and analyzed the adverse events related to SZC obtained from the FAERS and VigiAccess databases, with a view to providing more reference information for clinical safe drug use.

## Materials and methods

### Data source

Data for this study were obtained from anonymized adverse event reports in the publicly available FAERS and VigiAccess databases. During the data collection and analysis process, all authors did not have access to information that could identify individual participants. In this study, OpenVigil 2.1 was used to collect ADE reporting data in the FAERS database. OpenVigil 2.1 (https://openvigil.sourceforge.net/) [20,21] is a pharmacovigilance platform for extracting FAERS-related data, which has been widely used and verified [22–24]. Using the generic name of “Sodium Zirconium Cyclosilicate” and the trade name of “Lokelma” as search terms, we obtained the ADE report of sodium zirconium cyclosilicate as primary suspect (PS) drug. The retrieval time is from the first quarter of 2004 to the third quarter of 2024. In addition, the VigiAccess database was also searched up to February 5, 2025 using the generic name of the drug.

### Data processing and standardization

ADE report information was standardized and classified by using the Medical Dictionary for Regulatory Activities (MedDRA) (version 27.0). Each ADE record will be assigned a preferred term (PT) and further classified into different systems according to the system organ class (SOC).

### Statistical analysis

The disproportionality analysis is a signal detection method based on a two-by-two contingency table (Table 1) with high sensitivity and is widely used for monitoring and signal mining of adverse drug reactions [25,26]. This study used the reporting odds ratio (ROR) method and the proportional reporting ratio (PRR) method in the disproportionality analysis to identify risk signals in ADE reports collected from the FAERS database. The formulas used for PRR and ROR calculation are shown in Table 2. ROR signals were defined as positive when the number of cases was ≥ 3 and the lower limit of the 95% confidence interval (CI) was > 1. PRR signals were defined as positive when the number of cases was ≥ 3, PRR ≥ 2 and X^2^ ≥ 4 [27–31]. In this study, ADE which meets both PRR and ROR criteria is considered as a positive signal. Statistical analysis and data visualization were performed using Microsoft Office Excel 2019, OmicShare Tools [32] and R software (v.4.4.2).

**Table 1:** 2×2 contingency table for disproportionality analysis.

	Drug(s) of interest	All other drugs	Total
Adverse event(s) of interest	a	b	a + b
All other adverse events	c	d	c + d
Total	a + c	b + d	a + b + c + d

“a” represents the number of reports with adverse events of interest of the drugs of interest, “b” represents the number of reports with adverse events of interest of all other drugs, “c” represents the number of reports with all other adverse events of the drugs of interest, “d” represents the number of reports with all other adverse events of all other drugs.

**Table 2:** The formulas used for PRR and ROR calculation.

Algorithms	Equation	Criteria
ROR	ROR = ad/bc 95% CI = e${\;\;}^{\text{ln ROR}\pm 1.96(1/\text{a}+1/\text{b}+1/\text{c}+1/\text{d})0.5}$	a≥3, lower limit of 95% CI>1
PRR	PRR = a(c + d)/c(a + b) X^2^=[(ad - bc)^2^] (a + b + c + d)/[(a + b) (c + d) (a + c) (b + d)]	a ≥ 3, PRR ≥ 2, X^2^ ≥ 4

## Results

### Basic information reported by ADEs

A total of 1,384 ADE reports using SZC as the primary suspect in the FAERS database were collected via the OpenVigil 2.1 pharmacovigilance platform, and 1,518 ADE reports using SZC were retrieved from the VigiAccess database. There were significantly more males than females in both databases (FAERS: 48.19% vs 27.46%, p < 0.001; VigiAccess: 52.50% vs 30.83%, p < 0.001). In terms of patients’ age, patients with the age ≥ 75 years old were the most, followed by patients in the age group of 65–74 years old. In terms of geographical distribution, the majority of ADE reports originated from the Americas. The number of ADE reports associated with SZC showed a sustained upward trend over the reporting years, with a significant acceleration in the last two years. See Table 3 for basic information of the ADE reports.

**Table 3:** Basic information of ADE reports.

	FAERS (n = 1384)		VigiAccess (n = 1518)	
	*n*	%	*n*	%
Sex				
Female	380	24.46	468	30.83
Male	667	48.19	797	52.50
Unknown	337	24.35	253	16.67
Age				
<18	1	0.07	1	0.07
18-44	26	1.88	33	2.17
45-64	99	7.15	146	9.62
65-74	138	9.97	197	12.98
≥75	324	23.41	380	25.02
Unknown	796	57.51	759	50.00
Geographical distribution				
Africa	1	0.07	7	0.46
Americas	1092	78.90	1222	80.50
Asia	243	17.56	169	11.13
Europe	48	3.47	120	7.91
Unknown	0	0	0	0
Reporting year				
2018	6	0.43	0	0
2019	90	6.50	67	4.41
2020	110	7.95	115	7.57
2021	160	11.56	176	11.59
2022	264	19.08	296	19.50
2023	384	28.03	303	19.96
2024	388	28.03	14	0.92
2025	0	0	14	0.92

### SOC-level analysis for ADE reports

In FAERS database, the total number of reported cases of ADE associated with SZC was 2246, involving 26 SOCs. And the reported cases of general disorders and administration site conditions (714, 31.79%) ranked first, followed by gastrointestinal disorders (280, 12.47%) and investigations (260, 11.58%). In VigiAccess database, the total number of reported cases of ADE associated with SZC was 2555, involving 26 SOCs. The top three SOCs by number of reported cases were the same as in the FAERS database, namely, general disorders and administration site conditions (801, 31.35%), gastrointestinal disorders (324, 12.68%), and investigations (286, 11.19%). The SOCs involved in the two databases were the same, and the details of the SOC distribution of ADEs reported for SZC are shown in Tables 4 and 5.

**Table 4:** SOC distribution of ADEs reported for SZC in FAERS database.

SOC	PT	Case number	Percentage (%)
Gastrointestinal disorders	65	280	12.47
Investigations	61	260	11.58
Injury, poisoning and procedural complications	54	175	7.79
General disorders and administration site conditions	48	714	31.79
Nervous system disorders	40	98	4.36
Cardiac disorders	30	125	5.57
Metabolism and nutrition disorders	29	113	5.03
Infections and infestations	26	67	2.98
Renal and urinary disorders	25	101	4.50
Respiratory, thoracic and mediastinal disorders	22	54	2.40
Skin and subcutaneous tissue disorders	22	49	2.18
Psychiatric disorders	18	27	1.20
Musculoskeletal and connective tissue disorders	17	40	1.78
Neoplasms benign, malignant and unspecified (incl cysts and polyps)	17	24	1.07
Vascular disorders	16	31	1.38
Product issues	12	16	0.71
Surgical and medical procedures	8	12	0.53
Eye disorders	7	17	0.76
Hepatobiliary disorders	6	8	0.36
Ear and labyrinth disorders	5	7	0.31
Reproductive system and breast disorders	5	6	0.27
Blood and lymphatic system disorders	4	7	0.31
Immune system disorders	3	7	0.31
Social circumstances	3	5	0.22
Congenital, familial and genetic disorders	2	2	0.09
Endocrine disorders	1	1	0.04

**Table 5:** SOC distribution of ADEs reported for SZC in VigiAccess database.

SOC	PT	Case number	Percentage (%)
Gastrointestinal disorders	63	324	12.68
Investigations	71	286	11.19
Injury, poisoning and procedural complications	54	182	7.12
General disorders and administration site conditions	57	801	31.35
Nervous system disorders	40	115	4.50
Cardiac disorders	37	126	4.93
Metabolism and nutrition disorders	36	159	6.22
Infections and infestations	24	52	2.04
Renal and urinary disorders	27	114	4.46
Respiratory, thoracic and mediastinal disorders	20	53	2.07
Skin and subcutaneous tissue disorders	30	73	2.86
Psychiatric disorders	21	36	1.41
Musculoskeletal and connective tissue disorders	18	67	2.62
Neoplasms benign, malignant and unspecified (incl cysts and polyps)	14	20	0.78
Vascular disorders	18	39	1.53
Product issues	16	24	0.94
Surgical and medical procedures	10	22	0.86
Eye disorders	6	18	0.70
Hepatobiliary disorders	9	10	0.39
Ear and labyrinth disorders	4	5	0.20
Reproductive system and breast disorders	6	6	0.23
Blood and lymphatic system disorders	4	5	0.20
Immune system disorders	2	7	0.27
Social circumstances	4	8	0.31
Congenital, familial and genetic disorders	1	1	0.04
Endocrine disorders	2	2	0.08

### PT-level analysis for ADE reports

**Fig 1:**
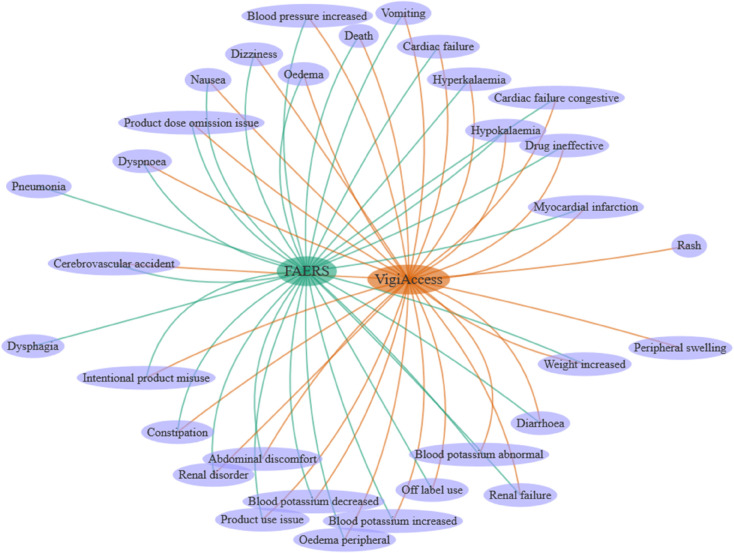
Network Venn diagrams of the top 30 PTs by number of reported cases in FAERS and VigiAccess databases.

In this study, the PTs involved in the ADEs reported for SZC were analyzed, with a focus on ADEs with high reporting frequency and high signal strength. The ADEs reported for SZC involved 546 PTs in FAERS (case number: 2246) and 594 PTs in VigiAccess (case number: 2555), respectively. Among the top 30 PTs in the number of reported cases, there were 28 coincident PTs in the two databases, as shown in Fig 1. The top 5 PTs reported in the FAERS database were death (n = 520, 23.15%), blood potassium increased (n = 81, 3.61%), constipation (n = 56, 2.49%), diarrhoea (n = 41, 1.83%), and oedema (n = 37, 1.65%), with the top 5 PTs reported in the VigiAccess database as death (n = 551, 21.57%), blood potassium increased (n = 83, 3.25%), diarrhoea (n = 61, 2.39%), hypokalemia (n = 56, 2.19%), and constipation (n = 53, 2.07%). Details of the top 30 PTs by number of reported cases are shown in Table 6.

**Table 6:** Top 30 PTs by number of reported cases.

	FAERS			VigiAccess		
	PT	Case number	Percentage (%)	PT	Case number	Percentage (%)
1	Death	520	23.15	Death	551	21.57
2	Blood potassium increased	81	3.61	Blood potassium increased	83	3.25
3	Constipation	56	2.49	Diarrhoea	61	2.39
4	Diarrhoea	41	1.83	Hypokalaemia	56	2.19
5	Oedema	37	1.65	Constipation	53	2.07
6	Cardiac failure	31	1.38	Oedema	37	1.45
7	Hypokalaemia	31	1.38	Nausea	34	1.33
8	Drug ineffective	27	1.20	Renal failure	31	1.21
9	Blood potassium abnormal	24	1.07	Off label use	28	1.10
10	Off label use	24	1.07	Product use issue	27	1.06
11	Renal failure	23	1.02	Hyperkalaemia	27	1.06
12	Weight increased	23	1.02	Blood potassium abnormal	26	1.02
13	Nausea	22	0.98	Drug ineffective	24	0.94
14	Blood pressure increased	21	0.93	Cerebrovascular accident	23	0.90
15	Hyperkalaemia	21	0.93	Cardiac failure congestive	22	0.86
16	Product use issue	21	0.93	Oedema peripheral	22	0.86
17	Cardiac failure congestive	19	0.85	Product dose omission issue	22	0.86
18	Cerebrovascular accident	19	0.85	Blood potassium decreased	21	0.82
19	Product dose omission issue	19	0.85	Weight increased	21	0.82
20	Abdominal discomfort	18	0.80	Dizziness	21	0.82
21	Myocardial infarction	17	0.76	Myocardial infarction	19	0.74
22	Pneumonia	17	0.76	Cardiac failure	18	0.70
23	Renal disorder	16	0.71	Rash	18	0.70
24	Vomiting	16	0.71	Abdominal discomfort	17	0.67
25	Blood potassium decreased	15	0.67	Vomiting	17	0.67
26	Dizziness	15	0.67	Peripheral swelling	17	0.67
27	Dysphagia	14	0.62	Blood pressure increased	17	0.67
28	Oedema peripheral	14	0.62	Dyspnoea	17	0.67
29	Dyspnoea	13	0.58	Intentional product misuse	16	0.63
30	Intentional product misuse	13	0.58	Renal disorder	15	0.59

**Fig 2:**
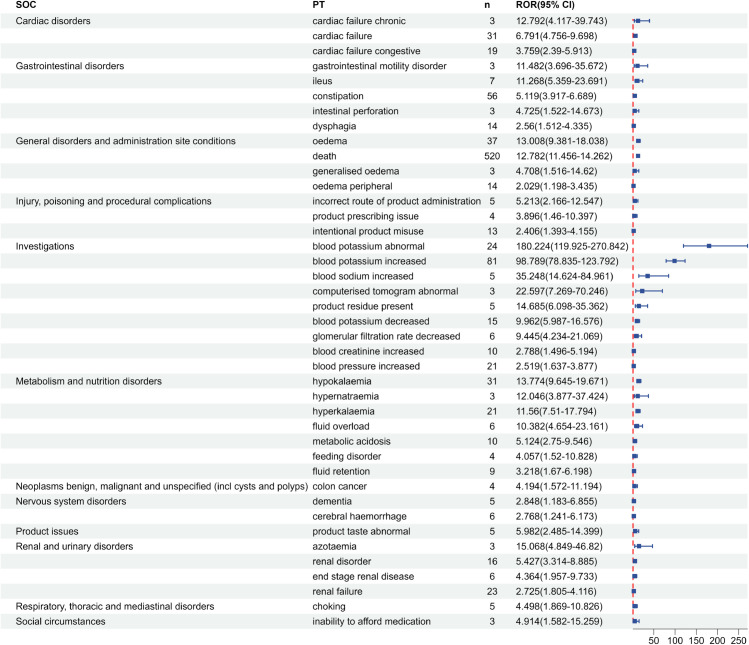
Positive signal detection results of SZC in FAERS database.

After screening by ROR and PRR methods, a total of 41 positive signals were screened out in FAERS database, as shown in Fig 2. The top 5 PTs by signal strength were blood potassium abnormal (n = 24, ROR = 180.224), blood potassium increased (n = 81, ROR = 98.789), blood sodium increased (n = 5, ROR = 35.248), computer tomography abnormal (n = 3, ROR = 22.597), and azotaemia (n = 3, ROR = 15.068).

**Fig 3:**
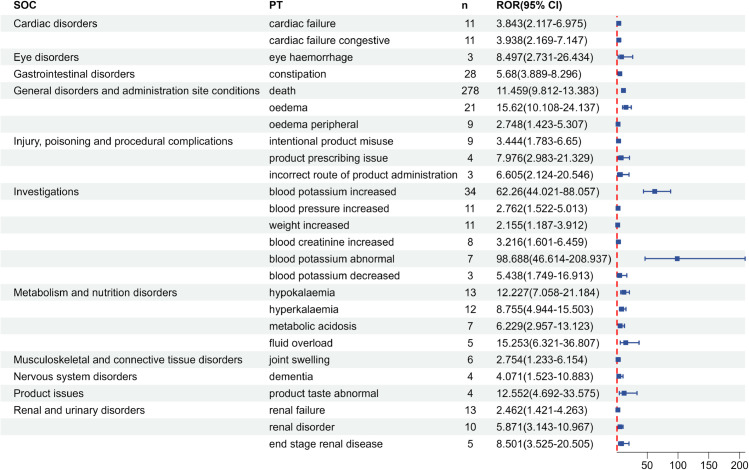
Positive signal detection results of SZC in male subgroup in FAERS database.

**Fig 4:**
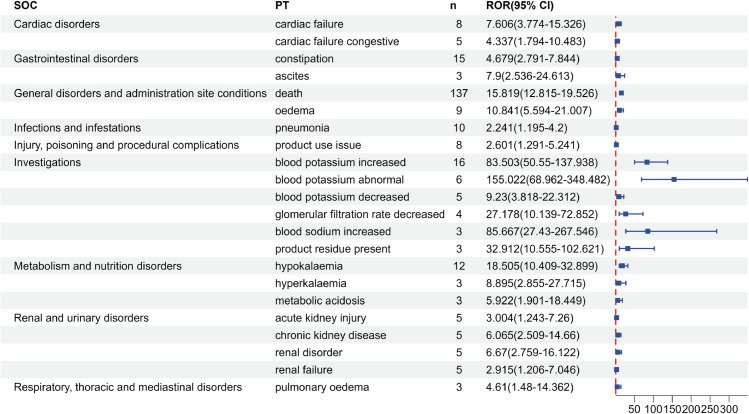
Positive signal detection results of SZC in female subgroup in FAERS database.

Subgroup analyses were conducted to explore sex-based differences in SZC-related adverse events, with comparison of positive signal PTs between male and female patients (Figs 3 and 4). In males, ADEs such as oedema, fluid overload, eye haemorrhage showed higher risk signals. In females, blood sodium increased, glomerular filtration rate decreased, ascites, and cardiac failure were identified as ADEs with higher occurrence risks.

## Discussion

Since its introduction to the market, SZC has emerged as the preferred regimen for the treatment of hyperkalemia. However, only a limited number of pre-marketing clinical trial studies have analyzed its safety profile, post-marketing pharmacovigilance studies based on real-world data remain insufficient. In this study, we analyzed the ADEs associated with SZC in real-world use by mining the data from the FAERS and VigiAccess databases, which provided more reference information for its safe clinical use.

This study found that the number of male patients with ADE reported in both databases exceeded that of female patients. This finding aligns with the results reported by Nilsson et al. [1] who conducted a study on the incidence of hyperkalemia within a large healthcare system. Their findings indicated that the incidence of hyperkalemia was lower in women. It is well known that hyperkalemia is prevalent among patients with CKD. A comprehensive review by Gilligan et al. [33] elucidated the prevalence and risk factors of hyperkalemia in patients with CKD, and pointed out that men were associated with higher serum potassium and a heightened risk of hyperkalemia in CKD. This gender difference in the prevalence of hyperkalemia may be a contributing factor to the observed sex disparities in reported ADEs.

In the FAERS and VigiAccess databases, the top 3 SOCs involved in ADEs reported for SZC were general disorders and administration site conditions, gastrointestinal disorders, and investigations, which were consistent with the common adverse drug reactions reported in the drug instructions. In addition, injury, poisoning and procedural complications, cardiac disorders, metabolism and nutrition disorders, nervous system disorders, and renal and urinary disorders were also the SOCs with the highest reported cases, and more attention should be paid to them in clinical application. In the PT-level analysis, most of the ADEs in the top 30 reported cases in the two databases were overlapping, proving the reliability of the results of this study to a certain extent. Among them, death, cardiac failure, weight increased, blood pressure increased, cardiac failure congestive, cerebrovascular accident, myocardial infarction, pneumonia, dizziness, dysphagia, and dyspnea were the ADEs with higher reporting frequency not included in the drug instructions, and they should be especially noted in clinical use.

In this study, according to the ROR method and PRR method, the ADEs in the FAERS database were screened to obtain positive signals, which involved 12 SOCs, including cardiac disorders, gastrointestinal disorders, general disorders and administration site conditions, injury, poisoning and procedural complications, investigations, metabolism and nutrition disorders, among others. Notably, signals such as cardiac failure chronic, cardiac failure, cardiac failure congestive, dysphagia, death, colon cancer, choking, dementia, cerebral hemorrhage, blood sodium increased, blood pressure increased, metabolic acidosis, and feeding disorder are not mentioned in the drug instructions, but these positive signals may be related to the patient’s own disease or edema-related events caused by SZC. Cardiac disorders such as cardiac failure chronic, cardiac failure, and cardiac failure congestive are ADEs not included in the drug instructions of SZC. In the Phase III clinical trial study of Roger et al. [34], there was a report about cardiac disorders caused by SZC, among 737 subjects, 158 subjects had serious adverse reactions, of which 10 patients had congestive cardiac failure and 4 patients had cardiac failure. The positive signals of gastrointestinal system diseases such as ileus and intestinal perforation are known severe gastrointestinal adverse events as traditional potassium-lowering drug SPS [35]. Although it is given a reminder in the precautions of SZC drug instructions, the risk situation is still unclear and more vigilance should be exercised in clinical use. Death is a positive signal of SZC, which has the highest number of reported cases. This may be due to the fact that patients with hyperkalemia are usually complicated with other diseases, such as severe heart failure and severe renal failure, and their baseline situation is poor. Multiple large-scale observational studies have shown that hyperkalemia itself is related to the increased risk of death [36–38]. Blood sodium increased and hypernatremia are two novel signals with high signal strength, which may be related to the potential sodium load existing in SZC (400mg sodium is contained in every 5g SZC). Therefore, capacity status should be monitored during use, and dietary sodium intake and diuretic amount should be adjusted in patients at risk of capacity overload [7,39]. In sex-based subgroup analyses, differences in adverse event profiles were observed. Beyond differences in signal strength for shared ADEs, sex-specific analyses revealed distinct positive signals unique to each sex. Notably, male-specific signals included eye haemorrhage and dementia, which were not detected in females. Conversely, females exhibited unique risk signals such as ascites, pulmonary oedema, and pneumonia absent in males. Although direct mechanistic evidence remains limited and requires further validation, these sex-divergent risk signals provide novel insights for pharmacovigilance and adverse reaction monitoring.

In our signal detection analysis based on the FAERS database, several positive signals were consistent with prior findings by Yu et al. [40], including cardiac failure congestive, cardiac failure chronic, ileus, intestinal perforation, blood sodium increased, hypernatremia, death. Utilizing the latest data from two databases for cross-validation, our study confirmed previously reported ADEs associated with SZC while also identified several new unreported signals of potential clinical significance, such as colon cancer, dementia, cerebral hemorrhage, choking, dysphagia, and feeding disorders. Special attention should be paid to these risks in clinical practice.

This study has several limitations. Both the FAERS database and VigiAccess database are spontaneous reporting systems for adverse events. As is well recognized, such pharmacovigilance databases are subject to biases, including underreporting, duplicate entries, inaccurate or incomplete information, and other factors that may affect the analysis. In this study, OpenVigil 2.1 platform was used to mine and analyze the data of FAERS. In OpenVigil 2.1, the cleaned data were only loaded with reports with complete case information, which sacrificed the original sample size to a certain extent but ensured high data quality [20]. Although the findings of large-scale data mining provide more reference information for safe medication use, the mining results can only show that there is a statistical correlation between drugs and the detected signals, and the exact causal relationship needs further clinical verification. Future research should be grounded in the findings of big data mining, and more high-quality clinical studies should be conducted on the focused adverse reactions to identify the occurrence of relevant adverse events in clinical use.

## Conclusion

This study conducted a comprehensive analysis of SZC by mining real-world adverse drug event databases, systematically evaluating high frequency and high signal strength adverse events to enhance clinical safety awareness. The research not only confirmed known adverse reactions but also identified previously unreported safety signals not mentioned in the drug instructions. These findings provide clinicians with more comprehensive safety references, particularly highlighting the importance of monitoring for these newly detected adverse reactions in clinical practice. In clinical use, healthcare professionals need to pay special attention to monitoring patients’ blood sodium levels, preventing cardiac risks such as chronic heart failure and gastrointestinal system risks such as ileus and intestinal perforation, as well as other risk signals detected. Pharmacovigilance research based on real-world data is imperative for providing recommendations for clinical decision-making and improving medication safety for patients.
